# Identification of methylation-regulated genes modulating microglial phagocytosis in hyperhomocysteinemia-exacerbated Alzheimer’s disease

**DOI:** 10.1186/s13195-023-01311-9

**Published:** 2023-10-03

**Authors:** Xianwei Wang, Lu Liu, Xiaohua Jiang, Jason Saredy, Hang Xi, Ramon Cueto, Danni Sigler, Mohsin Khan, Sheng Wu, Yong Ji, Nathaniel W. Snyder, Wenhui Hu, Xiaofeng Yang, Hong Wang

**Affiliations:** 1 Center for Metabolic Disease Research, Department of Cardiovascular Science, Lewis Kats School of Medicine, Temple University, MERB, Room 1060, 3500 N. Broad Street, Philadelphia, USA; 2 Key Laboratory of Cardiovascular Disease and Molecular Intervention, Nanjing Medical University, Nanjing, 211166 Jiangsu China

**Keywords:** Alzheimer’s, Hyperhomocysteinemia, Microglia, Phagocytosis, Hypomethylation, Amyloid β

## Abstract

**Background:**

Hyperhomocysteinemia (HHcy) has been linked to development of Alzheimer’s disease (AD) neuropathologically characterized by the accumulation of amyloid β (Aβ). Microglia (MG) play a crucial role in uptake of Aβ fibrils, and its dysfunction worsens AD. However, the effect of HHcy on MG Aβ phagocytosis remains unstudied.

**Methods:**

We isolated MG from the cerebrum of HHcy mice with genetic cystathionine-β-synthase deficiency (*Cbs*^*−/−*^) and performed bulk RNA-seq. We performed meta-analysis over transcriptomes of *Cbs*^*−/−*^ mouse MG, human and mouse AD MG, MG Aβ phagocytosis model, human AD methylome, and GWAS AD genes.

**Results:**

HHcy and hypomethylation conditions were identified in *Cbs*^*−/−*^ mice. Through *Cbs*^*−/−*^ MG transcriptome analysis, 353 MG DEGs were identified. Phagosome formation and integrin signaling pathways were found suppressed in *Cbs*^*−/−*^ MG. By analyzing MG transcriptomes from 4 AD patient and 7 mouse AD datasets, 409 human and 777 mouse AD MG DEGs were identified, of which 37 were found common in both species. Through further combinatory analysis with transcriptome from MG Aβ phagocytosis model, we identified 130 functional-validated Aβ phagocytic AD MG DEGs (20 in human AD, 110 in mouse AD), which reflected a compensatory activation of Aβ phagocytosis. Interestingly, we identified 14 human Aβ phagocytic AD MG DEGs which represented impaired MG Aβ phagocytosis in human AD. Finally, through a cascade of meta-analysis of transcriptome of AD MG, functional phagocytosis, HHcy MG, and human AD brain methylome dataset, we identified 5 HHcy-suppressed phagocytic AD MG DEGs (Flt1, Calponin 3, Igf1, Cacna2d4, and Celsr) which were reported to regulate MG/MΦ migration and Aβ phagocytosis.

**Conclusions:**

We established molecular signatures for a compensatory response of Aβ phagocytosis activation in human and mouse AD MG and impaired Aβ phagocytosis in human AD MG. Our discoveries suggested that hypomethylation may modulate HHcy-suppressed MG Aβ phagocytosis in AD.

**Supplementary Information:**

The online version contains supplementary material available at 10.1186/s13195-023-01311-9.

## Background

Alzheimer’s disease (AD) is the most common form of dementia worldwide, accounting for 60–70% of dementia cases, and is also a leading cause of death, ranking seventh globally and sixth in the United States (US) [1–3]. The hallmark features of AD neuropathology are the accumulation of amyloid β (Aβ) and the formation of neurofibrillary tangles [4]. Approximately 90% of individuals with clinically diagnosed AD had Aβ plaques based on positron emission tomography imaging [5]. A recently FDA-approved AD drug lecanemab is a humanized monoclonal antibody binding to soluble protofibrillar Aβ to promote Aβ removal by microglial (MG) phagocytosis [6] and is targeted for early-stage AD treatment [7]. Currently, there is no effective therapy for late-stage AD.

Pathological Aβ peptides are generated through the cleavage of the amyloid precursor protein (APP) by β- and γ-secretase enzymes [8]. Aβ aggregates over time to form oligomers, protofibrils, and fibrils/plaques. The accumulation of Aβ is a pivotal event in the development of AD, as it initiates a cascade of detrimental effects [8]. MG is responsible for 70% Aβ fibril uptake [9], primarily through MG receptor-mediated phagocytosis [10]. MG are brain-resident macrophages (MΦ) crucial in maintaining central nervous system homeostasis [11]. Therefore, research to discover the mechanisms and molecular targets responsible for Aβ generation and MG Aβ phagocytosis may lead to identifying therapeutic targets for late-stage AD.

Multiple epidemiological studies have established hyperhomocysteinemia (HHcy) as an independent risk factor for AD [12]. HHcy is a common metabolic disorder that occurs in 5–7% of the general population and up to 35% of the elderly population [13, 14]. In older adults, moderately elevated Hcy levels were associated with a 1.2-to-2.5-fold increased risk of developing dementia [12]. We [15–20] and others [21–24] have demonstrated a causative role and related mechanisms for HHcy in atherosclerosis, inflammatory monocyte (MC)/MΦ differentiation, and chronic kidney disease. The effect of HHcy on Aβ generation was tested in APP/PS1 transgenic AD mice with heterozygous dominant cystathionine-β-synthase (*Cbs*) mutants [25] and Tg2576 transgenic AD mice on a high methionine diet [26]. These studies reported that HHcy did not affect the levels or activity of β-secretase or its product, sAPPβ [25, 26]. Therefore, HHcy may not affect Aβ generation. The impact of HHcy on MG function and Aβ phagocytosis has not been well studied.

We hypothesize that HHcy impairs MG Aβ phagocytotic function via hypomethylation-related mechanism, which contributes to Aβ accumulation and exacerbated AD neuropathology in HHcy. We were the first to demonstrate that cellular hypomethylation via S-adenosylhomocysteine (SAH) accumulation is the primary biochemical mechanism in HHcy disease [27, 28]. This was supported by the concepts that SAH is an endogenous competitive inhibitor of S-adenosylmethionine (SAM) for methyltransferases and that the SAM/SAH ratio indicates cellular methylation status [29]. We described that HHcy induces DNA hypomethylation on the promoters of cyclin A and CD40 [19, 30], which led to endothelial cell growth suppression and inflammatory MC differentiation in HHcy conditions and chronic kidney disease. Recently, through a comprehensive large database analysis of 35 diseases, we concluded that the homocysteine-methionine (HM) cycle is a metabolic sensor system that modulates SAM/SAH-dependent methylation [31]. In addition, an increase of SAH accumulation was observed in the brain of *Mthfr*^*−*/*−*^ mice with moderate HHcy, which is associated with brain DNA hypomethylation [32]. Altered DNA and histone methylation have been suggested as a mechanism underlying AD development and neuropathology [33]. Differentially methylated DNA positions were identified in non-neuronal cell types, especially MG, and associated with AD pathology in human [34]. Therefore, HHcy-modulated DNA methylation in MG may play a critical role in the cause of AD development.

In this study, we performed a comprehensive meta-analysis over publicly available AD databases and an intensive literature search to identify molecular targets responsible for MG Aβ phagocytosis commonly in human and mouse AD. In addition, we conducted transcriptome analysis in MG isolated from HHcy mice with deficient *Cbs*. This combinatory effort led us to identify molecules potentially mediating HHcy-suppressed Aβ phagocytosis in AD MG.

## Methods

### HHcy mice

To create a HHcy model, we utilized the Tg-hCBS *Cbs*^*−/−*^ mice, which raised plasma Hcy levels up to 179.5 μM. The *Cbs*^*−/−*^ mice were generated following a previously described method [15, 35], in which a human CBS transgene (Tg-hCBS) was introduced under the control of a Zn-inducible metallothionein promoter to avoid neonatal lethality in the mice. The mice were fed a standard rodent chow diet and were euthanized at 18 weeks of age to collect blood and brain tissue. Eighteen mice from each group, *Cbs*^*−/−*^ mice and control, were used in this study for MG bulk RNA-seq analysis. The Temple University Institutional Animal Care and Use Committee approved all protocols involving mice.

### Measurement for Hcy, SAM, and SAH

Mouse blood collected in 1 mM ethylenediaminetetraacetic acid (EDTA)-coated tubes was centrifuged to obtain plasma, which was directly used for metabolite measurement. To measure tissue SAM and SAH, the cortex was harvested from mice, weighed, and homogenized with 0.4 M perchloric acid in a volume of 9 μl per 1 mg of tissue. After centrifugation, the resulting cortex extract was batched into 50 μl aliquots and stored at − 80 °C. For Hcy measurement, 50 mg of mouse cortex was collected. Liquid chromatography-electrospray ionization tandem mass spectrometry was used to determine the levels of Hcy, SAM, and SAH, following previously described methods [17].

### Mouse brain single cell suspension

Mouse brain cells were prepared using published protocols with modifications [36]. Briefly, one mouse cerebrum was finely minced with surgical scissors and digested in 2 ml of digestion mix containing 1 mg/ml collagenase D (Sigma-Aldrich, cat. no. 11088858001) and 50 μg/ml DNAse I (Sigma-Aldrich, cat. no. 10104159001) in RPMI 1640 medium, followed by incubation in a shaker at 37 °C for 30 min. The sample was pipetted onto a 100-μm nylon mesh strainer on a 50-ml tube, gently mashed using a glass pestle, and washed with 4 ml of fresh RPMI. This process was repeated two times. The sample was collected in a 15-ml conical tube and centrifuged at 256 × g for 5 min at 4 °C. The resulting cell pellets were resuspended in 8 ml of ice-cold 40% Percoll and carefully overlayed with 3 ml of 1 × HBSS, then centrifuged at 514 × g for 20 min at 4 °C (adjusting accelerating and braking value to 0). The top layer, which contained myelin debris, was carefully aspirated. The remaining materials were transferred into a new 50-ml tube, washed with 10 ml of ice-cold 1 × HBSS, and centrifuged at 348 × g for 10 min at 4 °C. Mouse brain cell pellets were then resuspended in 0.5 ml of 1640 medium for further experiments such as FACS and MG purification.

### Mouse brain MG purification with anti-CD11b conjugated microbeads

To prepare the anti-CD11b microbeads, 25 μl of sheep anti-rat Dynabeads (Invitrogen™) were washed three times with 0.75 ml of RPMI 1640 medium using a magnetic scaffold, allowing 1 min for the beads to settle each time. The Dynabeads were then suspended in 0.5 ml of serum-free 1640 medium and incubated overnight at 4 °C on a rotator (continuous rotation at 8 RPM) with 8 μl of rat anti-mouse CD11b (BD Pharmingen™, cat. no. 553308). After incubation, the anti-CD11b-conjugated microbeads were washed twice, resuspended in 0.5 ml of 1640 medium, and mixed with 0.5 ml prepared mouse brain cells. The mixture was incubated on a rotator for 30 min at 4 °C and then placed on a magnetic scaffold for 1 min, after which the supernatant was removed. The resulting MG-bead mixtures from six mice were pooled as one sample and washed with PBS before being used for RNA purification.

### Bulk RNA sequencing for MG from HHcy mice

Mouse MG were purified from the brain of *Cbs*^*−/−*^ and control mice using anti-CD11b microbeads. MG-bead mixtures from 6 mice in each group were pooled as one sample and directly used for total RNA extraction using the Monarch® Total RNA Miniprep Kit (#T2010s). Total RNA samples (500–800 ng/sample) were sent to Azenta/Genewiz for quality control, cDNA library preparation, and sequencing with specific configurations (Illumina HiSeq, 2 × 150 bp configuration, single index, per lane). Bulk RNA sequencing was performed for 3 samples in both groups, each pooled from 6 mice.

### RNA-Seq data analysis

We used Kallisto version 0.45 to align the reads in the raw RNA sequencing data against the mouse reference transcriptome (Hg38), using the classified information available (https://useast.ensembl.org/info/data/ftp/index.html). Genes with less than one count per million reads in at least three samples were filtered out, resulting in 16,859 normalized genes. The analysis was conducted in statistical computing environment R, using the Bioconductor suite of packages and RStudio (version 1.4.1717), which was described previously [37]. Differentially expressed genes (DEG) between the *Cbs*^*−/−*^ and control mice were identified using a criteria of fold change (FC) greater than or equal to 1.5 and a p-value less than 0.05 (FC > 1.5 and a *p*-value < 0.05). A volcano plot was used to visually represent the statistical significance and magnitude of DEG change.

### Identification of DEGs in human and mouse AD MG datasets

To identify the molecular targets mediating MG dysfunction in human and mouse AD, we analyzed published human and mouse AD MG datasets. Four human AD datasets were collected: (1) 75,060 single-nucleus (sn) transcriptomes from the prefrontal cortex of 24 control and 24 age-matched individuals with AD pathology [38], (2) 13,214 sn transcriptomes from the entorhinal cortex of 6 control and 6 AD patients [39], (3) 131,239 sn transcriptomes from the middle frontal neocortex of 9 control and 6 AD patients [40], (4) transcriptomes of MG sorted from the superior frontal gyrus of 15 control and 10 AD subjects [41]. Seven mouse AD MG datasets were collected: (1) bulk RNA-seq of MG from cortex of APPswe/PS1dE9 AD mice [42], (2&3) bulk RNA-seq of MG from cortex of PS2APP AD mice [43, 44], (4) bulk RNA-seq of MG from cortex of 5XFAD [45], (5) 73,419 sn transcriptomes from the cortex of 5XFAD mice [46], (6) 8016 single-cell (sc) transcriptomes in sorted CD45^+^ immune cells from the cortex of 5XFAD mice [47], (7) 15,041 sc transcriptomes in CD11b^+^ myeloid cells from the forebrain of APP/PS1 mice [48]. All above AD MG transcriptomes were reanalyzed to select the DEG using identical criteria applied in our own RNA-seq data analysis (FC > 1.5 and an adjusted *p*-value < 0.05). AD DEGs not in agreement across datasets were excluded. AD DEGs identified in all 4 human MG datasets were accepted but only DEGs discovered in at least 2 mouse MG datasets were identified as AD MG DEGs, which were used for further analysis. The identified AD MG DEGs were assigned the average FC value of each gene in the human or mouse AD MG datasets.

### *Establishment of mouse Aβ*^+^*MG DEGs, MG genetic modifiers of phagocytosis, endocytosis-related gene list, and identification of phagocytic AD MG DEGs*

To identify genes involved in Aβ phagocytosis in MG, we employed two Aβ phagocytosis functional datasets. The first one is a single-cell transcriptome which was established by comparing amyloid plaque-containing (Aβ^+^) and non-containing MG from the cerebrum of AD 5XFAD mice [49]. Heterozygous 5xFAD transgenic mice were injected intraperitoneally with florescent Methoxy-X04 which can cross the blood brain barrier and conjugate to Aβ plaques. Aβ^+^ and Aβ^−^ MG were sorted from cerebrum single-cell preparation and subjected to sc RNA-seq. Mouse Aβ^+^ MG DEGs were recognized by using identical criteria applied in our own RNA-seq data analysis (FC > 1.5 and an adjusted *p*-value < 0.05). The second Aβ phagocytosis functional dataset identified 286 MG genetic modifiers of phagocytosis by CRISPR-Cas9 knockout screening in phagocytic Cas9-MG-derived cell line BV2 infected with CRISPR-sgRNA library [50]. Additionally, we generated a gene list for endocytosis-related genes involved in the biological processes of endocytosis, exocytosis, and transcytosis by searching public Mouse Genome Informatics (https://www.informatics.jax.org/vocab/gene_ontology/GO:0006897) and Gene Set Enrichment Analysis (http://www.gsea-msigdb.org/gsea/msigdb/search.jsp) websites. We compiled a dataset consisting of 2172 endocytosis-related genes from various sources such as Gene Ontology (GO), Kyoto Encyclopedia of Genes and Genomes (KEGG), Reactome, and WikiPathways. Finally, phagocytic AD MG DEGs were identified by sequentially overlapping the endocytosis-related gene list, mouse Aβ^+^ MG DEGs, and MG genetic modifiers of phagocytosis with the above-identified human and mouse AD MG DEGs.

### Identification of phagocytic AD MG differentially methylated and expressed genes (DM/EG)

To establish the connection between AD genes and DNA methylation, as we proposed that hypomethylation is a key biochemical mechanism underlying HHcy disease, we examined AD DNA methylation and transcriptome datasets collected in an epigenome-wide meta-analysis involving prefrontal cortex from 1030 healthy controls and AD patients [51]. We selected 559 DM/EGs in human AD which were associated with altered RNA expression. These human AD DM/EGs were then used to perform overlap analysis with the above-identified 3 gene sets (377 mouse Aβ^+^ MG DEGs, 286 MG genetic modifiers of phagocytosis, and 215 endocytosis-related AD MG DEGs) to identify phagocytic Aβ^+^, phagocytic modifier, and endocytic-related AD MG DM/EGs. All were termed as phagocytic AD MG DM/EGs.

### Identification of GWAS-mapped AD MG DEGs

To generate a human GWAS-mapped AD gene list, we performed an intensive literature search in PubMed (https://pubmed.ncbi.nlm.nih.gov/). A total of 2016 articles were refined by imputing keywords “Alzheimer’s Disease” AND “GWAS” and carefully reviewed to 15 articles published between January 01, 2010, and May 31, 2022. A total of 431 human GWAS-mapped AD genes were extracted and overlapped with the above-identified 2 gene sets (409 human AD MG DEGs and 777 mouse AD MG DEGs) to identify GWAS-mapped AD MG DEGs in humans and mice. Expression profile of these genes in human AD and mouse AD models were established using databases of human and mouse AD. GWAS-mapped phagocytic AD MG DEGs were identified by overlapping identified human GWAS-mapped AD genes with phagocytic AD MG DEGs.

### Analysis of canonical pathway and biological process

The above-identified *Cbs*^*−*/*−*^ MG DEGs from bulk RNA-seq were subjected to canonical pathway analysis by applying version 7.1 of Ingenuity Pathway Analysis (IPA) in the Ingenuity Systems (https://www.ingenuity.com), as we previously reported [52]. Relevant pathways were selected based on *p*-value and *z*-score. Positive *z*-scores indicate activation of a pathway, whereas negative scores indicate inhibition. Biological processes were analyzed for human and mouse AD MG DEGs, and GWAS-mapped AD MG DEGs by applying GO enrichment analysis in the Gene Ontology Consortium’s website (http://geneontology.org/). GO biological processes with a false discovery rate (FDR) value lower than 0.05 were accepted as significantly enriched in this study.

## Results

### Overall strategy

**Fig. 1:**
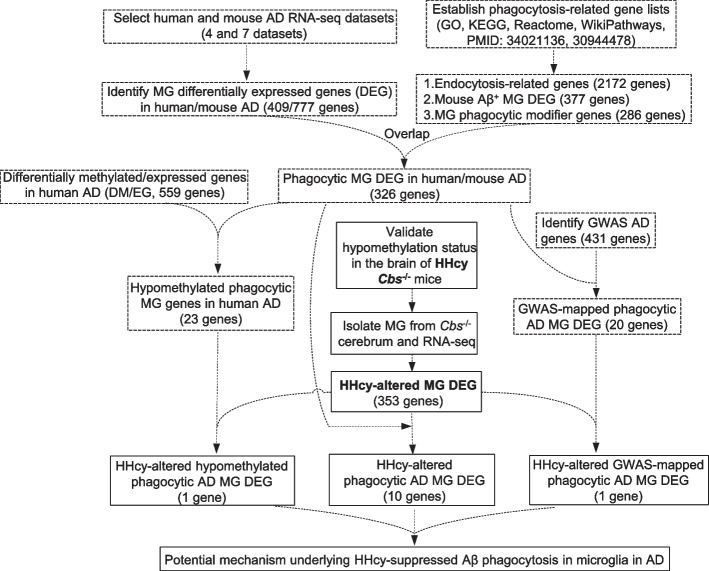
Overall strategy to identify potential mechanism underlying HHcy-suppressed Aβ phagocytosis in microglia in AD. We identified differentially expressed genes (DEG) in microglia (MG) from human and mouse Alzheimer’s disease (AD) RNA-seq datasets and performed overlap analysis with our established phagocytosis-related gene lists. This led to the discovery of 326 phagocytic MG DEGs in both human and mouse AD. In addition, we searched a human AD cortex methylome dataset and found 23 hypomethylated phagocytic MG genes in human AD. Furthermore, we performed a PubMed search to identify 431 AD genes from genome-wide association studies (GWAS) and found 20 GWAS-mapped phagocytic AD MG DEGs. To identify the gene targets that mediate the suppression of Aβ phagocytosis in microglia by HHcy in AD, we isolated MG from Cbs-/- and control mice and performed bulk RNA-seq. We used the identified 353 HHcy-altered MG DEGs to overlap with the three gene lists mentioned above, resulting in the identification of three final gene lists: HHcy-altered phagocytic AD MG DEGs (10 genes), HHcy-altered hypomethylated phagocytic AD MG DEGs (1 gene), and HHcy-altered GWAS-mapped phagocytic AD MG DEGs (1 gene), which was used to establish the hypothetic model for potential mechanism underlying HHcy-suppressed Aβ phagocytosis in microglia in AD. Aβ, amyloid beta; AD, Alzheimer’s disease; Cbs, cystathionine beta synthase; DEGs, differentially expressed genes; DMG, differentially methylated gene; MG, microglia; HHcy, hyperhomocysteinemia

This study aimed to identify gene targets that mediate the suppression of Aβ phagocytosis in MG by HHcy in AD. We employed comprehensive meta-analysis approaches for existing AD MG research databases and our transcriptome data for MG from severe HHcy mouse model (Fig. 1). We identified 326 phagocytic MG DEGs in human and mouse AD, 559 DM/EGs, 431 GWAS AD genes, and 353 HHcy-altered MG DEGs. Through multi-step overlapping analysis, we finally discovered a group of target genes that might be responsible for HHcy-exacerbated AD. These included HHcy-altered phagocytic AD MG DEGs (10 genes), HHcy-altered hypomethylated phagocytic AD MG DEGs (1 gene), and HHcy-altered GWAS-mapped phagocytic AD MG DEGs (1 gene).

### *Severe HHcy and suppressed methylation status in the plasma and cortex of Cbs*^*−/−*^* mice*

**Fig. 2:**
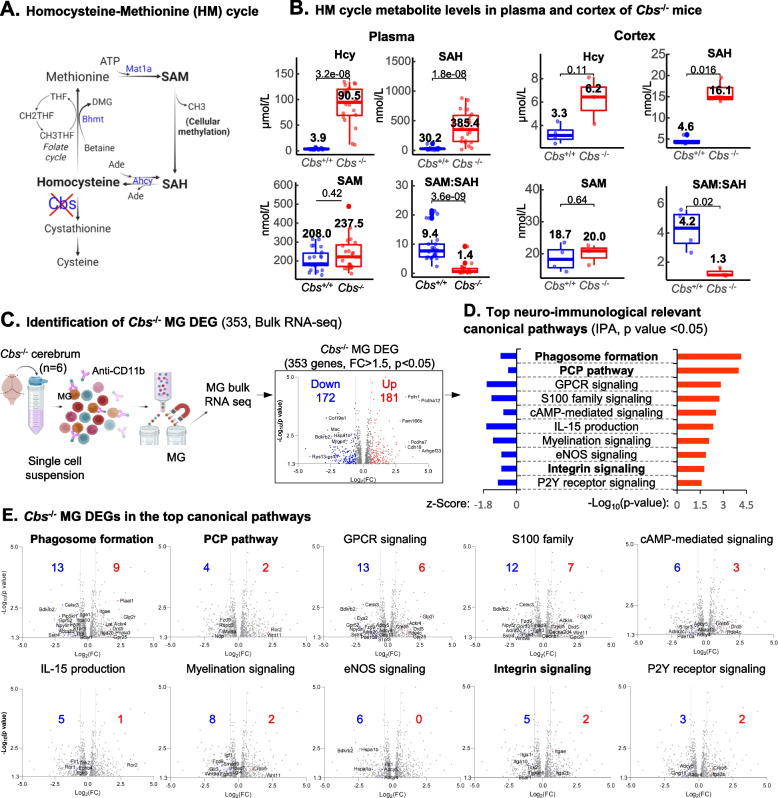
HM cycle metabolite levels and differentially expressed genes (DEG) identified in MG from HHcy *Cbs*-/- mice. **A** HM metabolic cycle. The production of universal methyl group donor SAM, methyltransferase inhibitor SAH and other major metabolites are indicated. **B** HM cycle metabolite in *Cbs*-/- mice. Metabolite levels in the HM cycle were evaluated in plasma and cortex from *Cbs*-/- and control mice using LC–ESI–MS/MS. Elevated levels of Hcy and SAH, as well as a reduced SAM/SAH ratio, were observed in the plasma and cortex of *Cbs*-/- mice. **C** Cerebrum RNA-seq and MG DEG identification. RNA sequencing was performed on MG isolated from the cerebrum of *Cbs*-/- and control mice using anti-CD11b Microbeads. DEGs in *Cbs*-/- MG were identified with a fold change > 1.5 and a *p*-value < 0.05. **D** Top neuro-immunological relevant canonical pathways. Pathways were identified using Ingenuity Pathway Analysis (IPA) based on 353 *Cbs*-/- MG DEG. The *p*-value and *z*-score are represented by red and blue bars, respectively. Positive *z*-scores indicate activation, whereas negative scores indicate inhibition of a pathway. **E** *Cbs*-/- MG DEGs in the top canonical pathways. DEGs involved in each of the top 10 canonical pathways in *Cbs*-/- MG are labeled in the volcano plots. MG migration and phagocytosis function related pathways are bolded. Ade, adenosine; *Cbs*, cystathionine beta synthase; DEG, differentially expressed gene; GPCR, G protein couple receptor; HHcy, hyperhomocysteinemia; IPA, ingenuity pathway analysis; LC–ESI–MS/MS, Liquid chromatography-electrospray ionization tandem mass spectrometry; Met, methionine; MG, microglia; PCP, planar cell polarity; SAH, s-adenosyl-L-homocysteine; SAM, s-adenosylmethionine

The HM metabolic cycle is also termed one-carbon metabolic cycle, which supplies methyl group for cellular methylation (Fig. 2A). CBS is the key enzyme responsible for Hcy clearance. Deficiency of the *Cbs* gene resulted in severe HHcy in *Cbs*^*−/−*^ mice, comparable to that seen in human HHcy [53]. In the plasma and cortex of *Cbs*^*−/−*^ mice, Hcy levels were increased to 90.5 μM and 6.2 μM (Hcy 3.9 μM and 3.3 μM in the control mice) (Fig. 2B). Methylation status is significantly suppressed in the plasma and cortex as SAM/SAH ratio is reduced to 1.4 and 1.3 in HHcy mice (9.4 and 4.2 in the control mice), exclusively due to the increase of SAH levels (385.4 nM and 16.1 nM in HHcy mice vs 30.2 nM and 4.6 nM in the control mice).

### *MG bulk RNA-seq in Cbs*^*−/−*^* mouse and 353 HHcy-altered MG DEG identification*

Through MG bulk RNA-seq analysis, we identified 353 MG DEGs in *Cbs*^*−/−*^ mice with fold change > 1.5 and *p* < 0.05 (Fig. 2C). A complete DEG list with fold changes details is provided in the Supplemental Table 1. We noticed that the top 10 neuro-immunological relevant canonical pathways were assigned with negative *z*-scores, suggesting a suppressive function direction (Fig. 2D), the inference further elaborated in Fig. 2E showing more downregulated DEGs in all these 10 pathways. Three pathways are relevant to MG migration and phagocytosis function, including phagosome formation, planar cell polarity (PCP), and integrin signaling.

### Identification of MG DEGs in human and mouse AD (AD MG DEGs) (human 409 and mouse 777 genes)

**Fig. 3:**
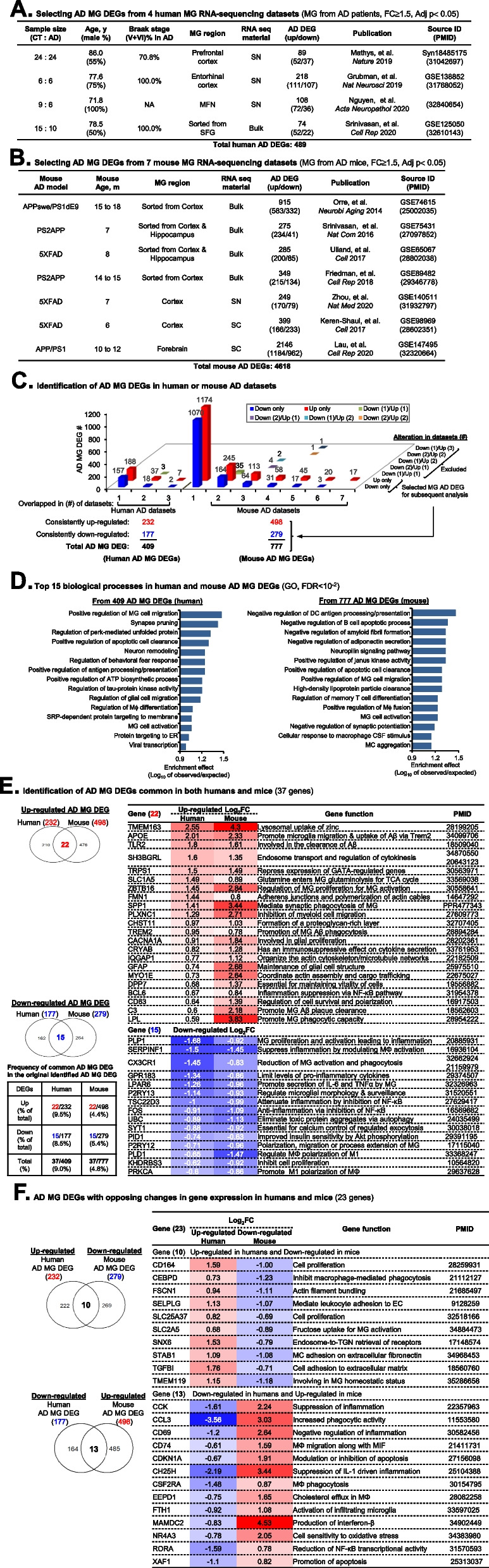
Identification of DEGs in MG from AD human and mouse. **A** Summary of four human MG RNA-sequencing datasets. DEGs in human MG from AD patients compared to healthy controls using 4 SN and bulk RNA-seq datasets. The criteria for selecting DEGs were FC ≥ 1.5 and adjusted *p*-value < 0.05. **B** Summary of seven mouse MG RNA-sequencing datasets. DEGs in MG from AD mice versus control were identified in 7 RNA-seq datasets, including SN, SC, and bulk RNA-seq studies. The same criteria for selecting DEGs were applied. **C** Selection of total AD MG DEGs. A total of 409 AD MG DEGs in human and 777 overlapped at least in two mouse datasets were selected, excluding those that had the opposite direction of gene expression alteration. **D** Top 15 biological processes. The top 15 biological processes for the selected human and mouse AD MG DEGs were identified using Gene Ontology analysis. Enrichment effect describes the strength of the process. **E** Overlapped AD MG DEGs common in both human and mouse. The study identified a total of 22 upregulated and 15 downregulated AD MG DEGs that overlapped in both human and mouse datasets. **F** The study identified 23 AD MG DEGs with opposing changes in gene expression in humans and mice. AD, Alzheimer’s disease; CSF, colony stimulating factor; CT, control; DC, dendritic cell; DEG, differentially expressed gene; ER, endoplasmic reticulum; FC, fold change; GO, gene ontology analysis; MFN, middle frontal neocortex; MG, microglia; MIF, macrophage migration inhibitory factor; NA, not available; SC, single cell; SFG, superior frontal gyrus; SN, single nucleus; TGN, trans-Golgi-network

Using identical selecting criteria (FC > 1.5 and an adjusted *p*-value < 0.05), we selected total 489 DEGs from 4 human MG RNA-sequencing datasets (89, 218, 108, and 74) and 4618 DEGs from 7 mouse MG RNA-sequencing datasets (915, 275, 285, 349, 249, 399, and 2146) as detailed in Fig. 3A and B. Five mouse AD models were utilized, all characterized by mutations in APP/PS1 and elevated levels of the Aβ1-42/Aβ1-42 ratio. These models included APPswe/PS1dE9, PS2APP, 5XFAD, and APP/PS1 mice. We identified 409 human AD MG DEGs (232 up/177 down) by excluding DEGs with opposing direction of RNA expression alterations (Fig. 3C and Supplemental Table 2). Considering the genetic similarity of mouse AD models, we only accepted DEGs discovered in at least 2 datasets and excluded DEGs with opposing directions. This led to identification of 777 mouse AD MG DEGs (498 up/279 down) (Fig. 3C and Supplemental Table 3).

### Identification of MG activation pathways in human and mouse AD MG

MG migration, differentiation, and activation pathways were recognized by GO functional enrichment analysis for the above-identified human and mouse AD MG DEGs (Fig. 3D). Other relevant pathways included neuron remodeling, synapse pruning, regulation of tau-protein kinase activity, regulation of behavioral fear response for human AD MG DEGs, and negative regulation of amyloid fibril formation, high-density lipoprotein particle clearance, negative regulation of synaptic potentiation, and regulation of T/B cell immunity for mouse AD MG DEGs.

### Identification of AD MG DEGs common in both humans and mice (37 genes)

For the justification of using mouse AD model for mechanistic research of human AD, we performed overlap analysis between human and mouse AD MG DEGs and identified 22 upregulated and 15 downregulated AD MG DEGs common in both humans and mice (Fig. 3E). As indicated in the table, most of these genes possess functions in MG migration, activation of phagocytosis, alternative polarization, and neuroinflammation. We noted the low frequency of common AD MG DEGs in the originally identified human and mouse AD MG DEGs (9.0% and 4.8%, respectively), indicating inconsistency between human AD and mouse AD models. Figure 3F showed the identified 23 AD MG DEGs with opposing changes in gene expression in humans and mice.

### Identification of phagocytic AD MG DEGs in human and mouse (326 genes)

**Fig. 4:**
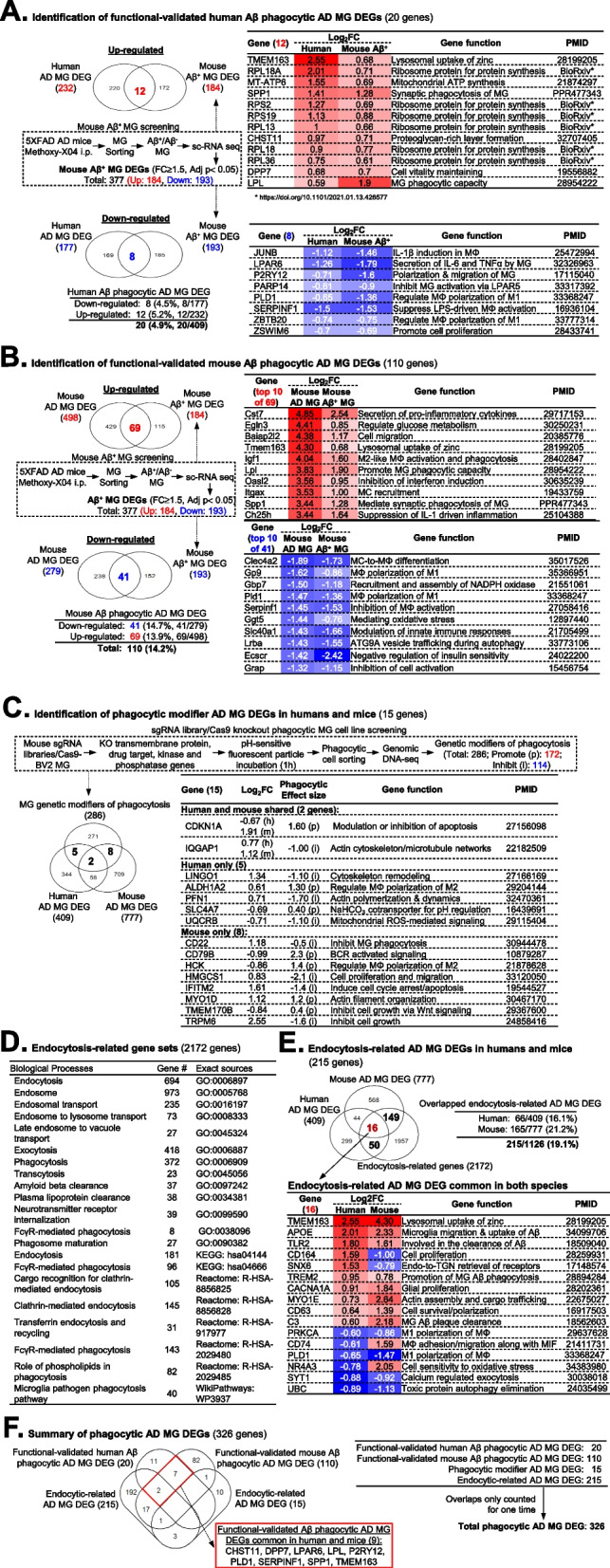
Identification of 326 phagocytic AD MG DEGs and relevant function. **A** Functional-validated human Aβ phagocytic AD MG DEGs. The embedded dashed box shows identification of 184 upregulated and 193 downregulated Aβ + MG DEGs in GSE165306. MG were isolated from the Cerebrum of AD 5xFAD tg mice injected with florescent Methoxy-X04 (MeX04) that labels Aβ plaque and sorted for Aβ + and Aβ − populations for further scRNA-seq analysis. The Aβ + MG DEGs were identified with FC ≥ 1.5 and adjusted *p*-value < 0.05 and overlapped with the previously identified functional-validated human AD MG DEGs. **B** The functional-validated mouse Aβ phagocytic AD MG DEGs were identified by overlapping the Aβ + MG DEG with the previously identified mouse AD MG DEGs using the same strategy in **A**. **C** Identification of phagocytic modifier AD MG DEGs. MG genetic modifiers of phagocytosis were identified by conducting a phagocytic genetic modifier screening using CRISPR-Cas9 for knockout of genes encoding membrane proteins, drug targets, kinases, or phosphatases in the BV2 cell line derived from phagocytic microglia (PMID: 30,944,478). The Venn diagram shows the overlap of BV2 cell phagocytic modifier genes with AD MG DEGs of human and mouse. **D** Gene sets related to endocytosis. A total of 2172 genes involved in biological processes of endocytosis, exocytosis, and transcytosis were collected from GO, KEGG, Reactome, and WikiPathways. **E** Overlapped endocytosis-related AD MG DEGs. The overlap analysis of 2172 endocytosis-related genes with 409 human and 777 mouse AD MG DEGs identified 16 overlapped endocytosis-related AD MG DEGs in human and mouse. **F** Total phagocytic AD MG DEG is summarized. Aβ, amyloid beta; AD, Alzheimer’s disease; DEG, differentially expressed gene; FC, fold change; i, inhibit; i.p., intraperitoneally; MeX04, Methoxy-X04; MG, microglia; MIF, macrophage migration inhibitory factor; p, promote; SC, single cell; TGN, trans-Golgi-network

We identified 20 functional-validated human Aβ phagocytic AD MG DEGs (12 up/8 down) and 110 functional-validated mouse Aβ phagocytic AD MG DEGs (69 up/41 down) by overlapping the 184 upregulated and 193 downregulated mouse Aβ^+^ MG DEGs selected from a mouse Aβ^+^ MG screening dataset [49] with the same AD MG DEGs (Fig. 4A and B, Supplemental Tables 4 and 5). We recognized 12 upregulated functional-validated human Aβ^+^ MG DEGs, including 6 ribosome proteins (RPL18A, RPS2, RPS19, RPL13, RPL18, RPL36), 3 Aβ clearance-related genes (TMEM163, SPP1, and LPL), 3 other genes (MT-ATP6, CHST11, DPP7), and 8 downregulated Aβ^+^ MG DEGs, all possessing MΦ activation and inflammation related function (Fig. 4A). In addition, among the top 10 of upregulated and downregulated functional-validated mouse Aβ phagocytic AD MG DEGs, we noted that the upregulated genes are mostly involved in MG phagocytosis, and the downregulated genes are mostly involved in MΦ differentiation and autophagy (Fig. 4B). The proportion of functional-validated phagocytic AD MG DEGs was lower in human AD (4.9%) compared to mouse AD (14.2%), suggesting limited or impaired MG Aβ phagocytosis in human AD. Supplemental Figure S1 further showed a total of 14 functional-validated human Aβ phagocytic AD MG DEGs with opposing changes in gene expression in human AD MG and mouse Aβ^+^ MG. However, none of the functional-validated mouse Aβ phagocytic AD MG DEGs were opposing changes in gene expression in mouse Aβ^+^ MG. In addition, we identified 15 phagocytic modifier AD MG DEG by overlapping 286 MG genetic modifiers of phagocytosis selected from a sgRNA library/Cas9 knockout phagocytic MG cell line screening dataset [50] with above-identified human and mouse AD MG DEG. Actin cytoskeleton network gene IQGAP1 was recognized as upregulated phagocytic modifier AD MG DEG in both species (Fig. 4C and Supplemental Table 6). By overlapping the 2172 endocytosis-related genes with the above-identified human and mouse AD MG DEG, we identified 215 endocytosis-related AD MG DEGs, including 66 in humans and 165 in mice (Fig. 4D,E, Supplemental Tables 7 and 8). Among the 16 endocytosis-related AD MG DEGs common in both species, we recognized 8 upregulated genes including 5 Aβ clearance-related genes (TMEM163, APOE, TLR2, TREM2, and C3), glial proliferation gene CACNA1A, actin assembly and cargo trafficking gene MYO1E, and cell survival/polarization gene CD63 and 4 downregulated genes including M1 polarization genes PRKCA and PLD1 and exocytosis and autophagy genes SYT1 and UBC, based on their consistent expression direction in both species. Finally, we obtained a total of 326 phagocytic AD MG DEG by combining functional-validated human/mouse Aβ phagocytic AD MG DEGs, phagocytic modifier AD MG DEGs, and endocytosis-related AD MG DEGs (Fig. 4F and Supplemental Table 9). We also found that 9 functional-validated Aβ phagocytic AD MG DEGs, out of 130, were shared in both humans/mice and functional Aβ phagocytosis model (CHST11, DPP7, LPAR6, LPL, P2RY12, PLD1, SERPINF1, SPP1, TMEM163).

### Identification of GWAS-mapped phagocytic AD MG DEGs (20 genes)

Through intensive literature search, we identified 431 human AD GWAS-mapped AD genes (Supplemental Figure S2A and Supplemental Table 10). After overlapping analysis with the above-identified human and mouse AD MG DEG, we discovered 43 GWAS-mapped AD MG DEGs (Supplemental Figure S2B), in which 17 were identified in human AD and 30 in mouse AD models. Their expression profiles in human and mouse AD models are presented in Supplemental Figures S2C and D. Interestingly, human and mouse AD shared 4 GWAS-mapped AD MG DEGs, 3 of which were upregulated in both human and mouse AD (TMEM163, APOE, TREM2), all possessing the phagocytic function. Interesting biological processes of these 43 GWAS-mapped AD MG DEGs were mostly related with MG/MΦ activation, Aβ clearance, and IGF-R signaling (Supplemental Figure S2E). Further overlapping analysis of human GWAS-mapped AD genes (431) with phagocytic AD MG DEGs (326) led to the identification of 20 GWAS-mapped phagocytic AD MG DEGs. Their phagocytosis and MG/MΦ activation functions were supported by published studies, and their determined subcellular localizations based on GeneCards are presented in Supplemental Figure S2F. Finally, we identified Igf1, as the only GWAS-mapped phagocytic AD MG DEG, which was reduced by 1.79-fold in MG isolated from our HHcy *Cbs*^*−*/*−*^ mice.

### Identification of hypomethylated phagocytic AD MG DM/EGs (23 genes)

**Fig. 5:**
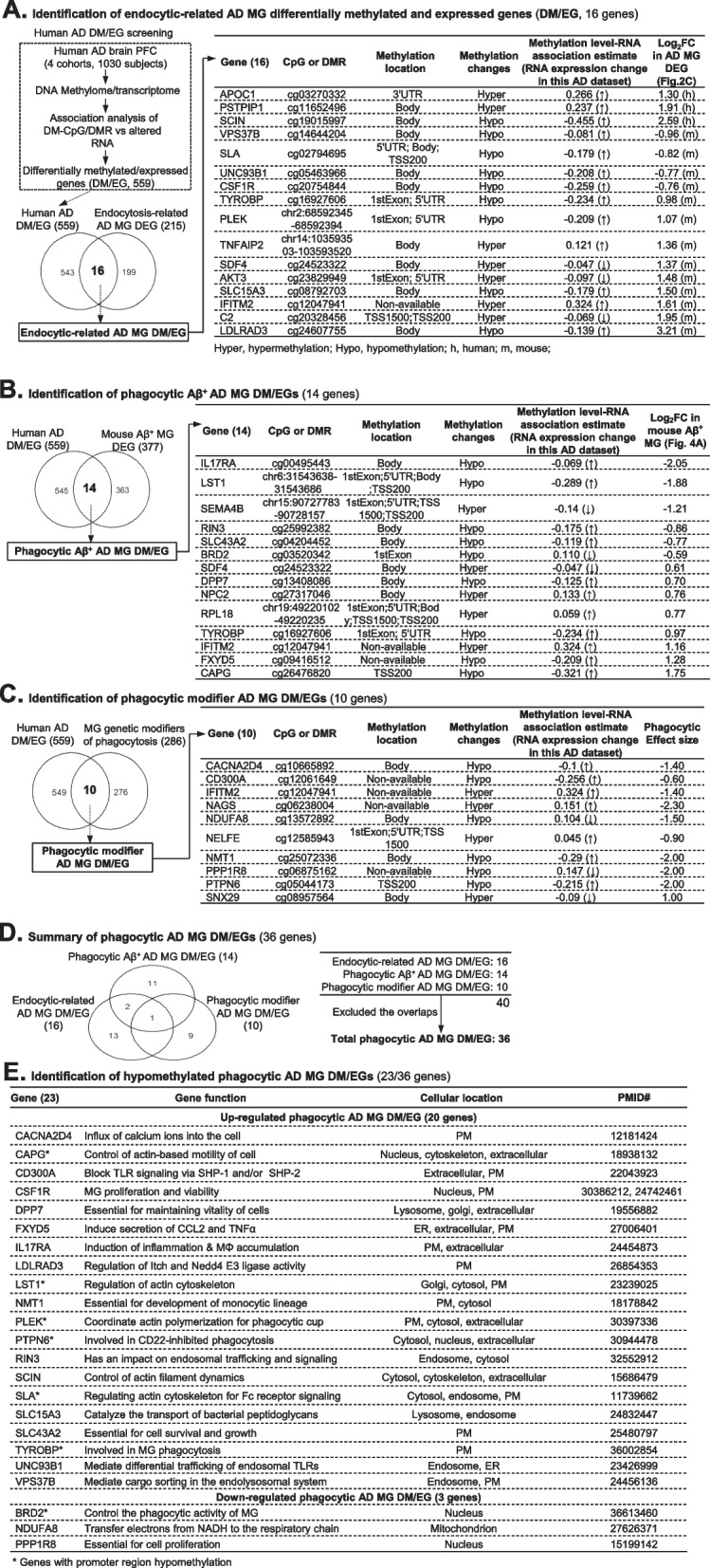
Identification of differentially methylated/expressed genes (DM/EG) in hypomethylated phagocytic AD MG. **A** Endocytic-related AD MG DM/EGs. From a whole-genome DNA methylation dataset (PMID: 33,257,653) in prefrontal cortex (PFC) samples from 1030 Alzheimer’s disease (AD) patients and healthy controls, we identified 559 DM/EG in AD by associating DM-CpG/DMR with altered RNA expression. A total of 16 endocytic-related DM/EGs were identified by overlapping these 559 human AD DM/EGs with 215 endocytosis-related AD MG DEGs. Table describes the epigenetic modification and expression details of these genes in both human and mouse AD MG. **B** We also identified 14 phagocytic Aβ + AD MG DM/EGs and **C** 10 phagocytic modifier AD MG DM/EGs, by overlapping the 559 human AD DM/EGs with 377 Aβ + MG DEGs and 286 phagocytic modifier genes, respectively. **D** A total of 36 phagocytic AD MG DM/EGs are summarized. **E** Function of 23 hypomethylated phagocytic AD MG DM/EGs. Among the 36 phagocytic AD MG DM/EG, 23 were hypomethylated associated with their gene upregulated (20 genes) and downregulated (3 genes) expression. Aβ, amyloid beta; AD, Alzheimer’s disease; DEG, differentially expressed gene; DMEG, differentially methylated expressed genes; DMR, differentially methylated regions; ER, endoplasmic reticulum; FC, fold change; h, human; m, mouse; MG, microglia; PFC, prefrontal cortex; PM, plasma membrane

We selected 559 human AD DM/EGs from published datasets [51] (Supplemental Table 11) and incorporated into our searching for methylation-regulated phagocytic AD MG DEGs. We identified 16 endocytic-related AD MG DM/EGs, 14 phagocytic Aβ^+^ AD MG DM/EGs, and 10 phagocytic modifier AD MG DM/EGs by a series of overlapping analysis with three phagocytosis-related datasets that we discovered in this study (Fig. 5A,B,C). The methylation changes on these genes, hyper- or hypo-methylation, were associated with RNA expression changes in the same AD dataset and compared with AD MG DEGs identified in this study (Fig. 2C). A total of 36 phagocytic AD MG DM/EGs were identified (Fig. 5D). A total of 23 hypomethylated phagocytic AD MG DM/EGs were identified (Fig. 5E). These genes mostly possess functions related with MG activation/phagocytosis, inflammation, and survival. Finally, we found 7 hypomethylated phagocytic AD MG DM/EGs which presented promoter hypomethylation changes in this human AD DM/EG screening study [51].

### Identification of HHcy-altered phagocytic AD MG DEGs (10 genes)

**Fig. 6:**
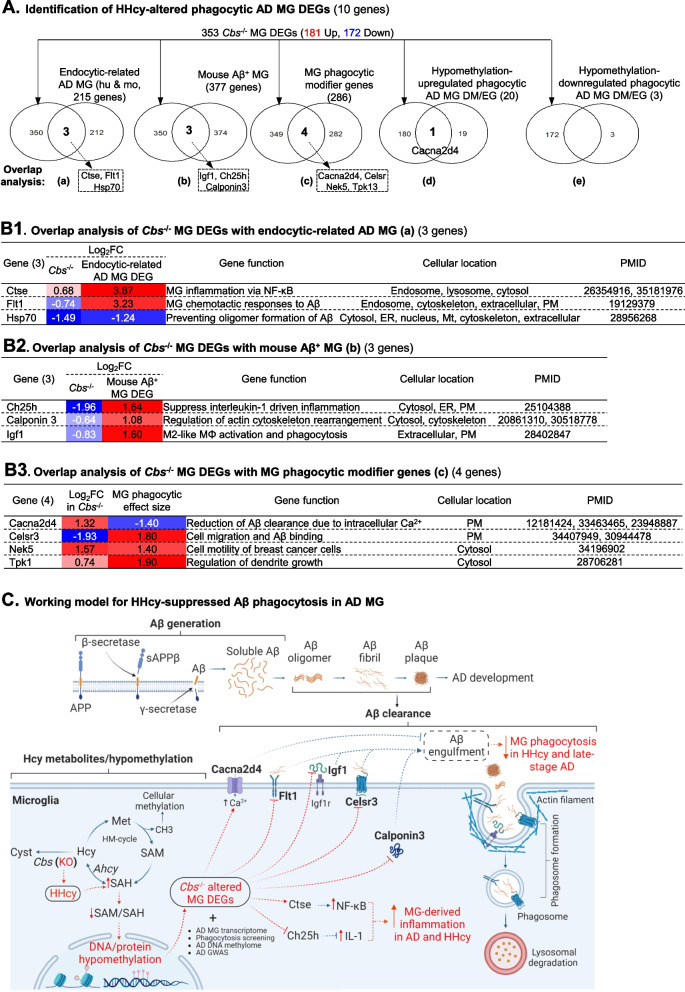
Identification of HHcy-altered phagocytic AD MG DEGs and working model for HHcy-suppressed MG Aβ phagocytosis. **A** Identification of HHcy-altered phagocytic AD MG DEGs. Through overlapping the *Cbs*-/- MG DEG with 5 phagocytic MG DEGs identified in this study, a total of 10 HHcy-altered phagocytic AD MG DEGs were determined. **B** Overlap analysis of *Cbs*-/- MG DEGs with other MG DEGs related with AD phagocytosis and hypomethylation. The tables display the gene function, cellular location, and expression changes observed in both *Cbs*-/- MG and AD-related MG models used in this analysis. **C** Working model for HHcy-suppressed Aβ phagocytosis in AD MG. The proposed model elucidates the process of Aβ generation and cellular clearance, and the impact of impaired HM cycle on histone/DNA hypomethylation, and potential molecular targets/signaling pathways underlying HHcy-suppressed Aβ phagocytosis in AD MG. Aβ, amyloid beta; AD, Alzheimer’s disease; *Cbs*, cystathionine beta synthase; Cyst, cystathionine; DEG, differentially expressed gene; DMG, differentially methylated genes; ER, endoplasmic reticulum; FC, fold change; HHcy, hyperhomocysteinemia; His, histone; HM, homocysteine-methionine; KO, knockout; MG, microglia; Mt, mitochondria; PM, plasma membrane

To identify genes potentially mediating HHcy-exacerbated AD, 353 *Cbs*^*−*/*−*^ MG DEGs were employed for overlapping analysis with the five category AD MG phagocytosis-related genes identified in this study and described as overlap analysis A-E (Fig. 6A). We identified 3 HHcy-altered phagocytic AD MG DEGs (Ctse, Flt1, Hsp70), 3 HHcy-altered Aβ^+^ MG DEGs (Ch25h, Calponin 3, Igf1), 3 HHcy-altered MG phagocytic modifier genes (Cacna2d4, Celsr3, Nek5, Tpk1), and 1 HHcy-altered hypomethylation-upregulated phagocytic AD MG DM/EG Cacna2d4, which was also identified as a HHcy-altered MG phagocytic modifier gene (Fig. 6B1,B2,B3). Therefore, we totally identified 10 HHcy-altered phagocytic AD MG DEGs. Based on literature search, 5 of these genes (Flt1, Calponin 3, Igf1, Cacna2d4, Celsr) were reported with experimentally confirmed functions related with MG/MΦ migration and phagocytosis, which was used to establish a model for potential mechanism underlying HHcy-suppressed Aβ phagocytosis in microglia in AD (Fig. 6C).

## Discussion

We conducted a comprehensive meta-analysis by combining available AD datasets, MG Aβ phagocytosis transcriptome, and our own transcriptome study of MG from HHcy *Cbs*^*−*/*−*^ mice. We identified 9 important gene lists, including (1) 353 HHcy-altered MG DEGs, (2&3) 409 human and 777 mouse AD MG DEGs, (4) 37 AD MG DEGs common in both humans and mice, (5) 130 functional-validated Aβ phagocytic AD MG DEGs (20 in human AD, 110 in mouse AD, and 9 overlapped in both species) reflecting compensatory MG phagocytosis activation in both species, (6) 14 functional-validated Aβ phagocytic AD MG DEGs representing impaired MG Aβ phagocytosis in human AD, (7) 20 GWAS-mapped phagocytic AD MG DEGs, (8) 23 hypomethylated phagocytic MG DM/EGs in human AD, and (9) 5 HHcy-suppressed phagocytic AD MG DEGs. We established models describing molecular signatures for MG Aβ phagocytosis functional changes in human and mouse AD, and HHcy.

### HHcy-SAH-related hypomethylation is associated with AD pathology in HHcy

We characterized HHcy and hypomethylation status in the plasma and brain cortex of *Cbs*^*−*/*−*^ mice (Fig. 2). These data provided strong evidence to support the hypothesis that HHcy and consequential SAH elevation-related hypomethylation may mediate HHcy-exacerbated AD. We were first to propose the HHcy-SAH-related hypomethylation theory supported by others [29, 54]. In *Cbs*^+/*−*^ mice fed a methionine-choline-folate-deficient diet, mild HHcy was associated with increased SAH, and SAM to SAH ratio was negatively correlated with DNA methylation [29]. In HHcy *Mthfr*^+/*−*^ mice fed with a high methionine/low folate diet, mild HHcy also was associated with increased SAH, decreased SAM to SAH ratio, and increased global DNA hypomethylation in the brain [54]. Numerous clinical and preclinical studies reported strong association of HHcy with cognitive impairment [12, 55]. HHcy AD mice displayed increased levels of brain Aβ peptides [25, 26]. Besides Aβ accumulation, hyperphosphorylated tau protein (neurofibrillary tangle) is another important feature of AD neuropathology. It was reported that tau phosphorylation and oligomerization was increased in HHcy condition in a human neuroblastoma M1C cells (Hcy > 100 μM treatment) and in a tauopathy TG4510 mice fed a folate deficient diet for 4 weeks (Hcy levels not mentioned) [56]. It was suggested that HHcy could promote N-homocysteinylation of tau by targeting lysine residues critical for their binding to β-tubulin in AD patients and rodent AD models [57]. Importantly, it was well-documented that tau protein is the downstream target of Aβ species and that Aβ plaques facilitate tau protein misfolding leading to its accumulation and neurofibrillary tangle formation [8]. All of these studies supported the role of HHcy in AD and the hypothesis that hypomethylation mediates HHcy-exacerbated AD.

### HHcy-SAH-related DNA hypomethylation may mediate MG phagocytosis suppression in AD

We discovered 353 DEGs in MG isolated from HHcy *Cbs*^*−/−*^ mice and 3 top pathways relevant to MG migration and phagocytosis (Fig. 2). Experimental evidence was reported to support phagosome formation pathway for phagocytosis [58], PCP pathway for neuronal migration [59], and β1 integrin signaling for fibrillar Aβ engulfment [60]. Recently, DNA methylation signatures of AD has been reported in an epigenome-wide association study using the human AD cortex, suggesting that AD neuropathology was associated with DNA methylation changes primarily in MG [34]. In addition, epigenetic modifications also were proposed for MG functional change in neurodegenerative disease [61]. Therefore, DNA hypomethylation may be a mechanism for impaired MG function. We previously hypothesized that Hcy metabolism is a key metabolic sensor system controlling methylation-regulated pathology [31]. We described that HHcy caused SAH accumulation, RAS c-terminal carboxyl hypomethylation and cyclin A cycle-dependent element suppressor binging site hypomethylation in cultured human primary endothelial cells leading to suppressed cell proliferation [27, 30] and CD40 promoter hypomethylation in inflammatory MCs [19]. The evidence above supports the hypothesis that HHcy-SAH-related hypomethylation, especially DNA hypomethylation, may be a critical mechanism compromising the ability of MG phagocytosis in AD pathology.

### DNA hypomethylation of 23 phagocytosis genes was identified in human AD MG

We found 23 hypomethylated phagocytic AD MG DM/EGs by screening the whole genome methylome of differentially methylated-CpG/region of human AD brain, in which PTPN6 DNA methylation levels were inversely correlated with its mRNA levels in human AD MG (Fig. 5). Since PTPN6 was reported to be critical in inhibiting MG Aβ phagocytosis [50], PTPN6 DNA hypomethylation and increased expression in AD MG may contribute to Aβ plaque accumulation and other neurodegenerative diseases. In contrast, BRD2 DNA methylation was positively correlated with its mRNA levels in human AD MG. BRD2 expression was associated with the increased phagocytic activity of MG cell line BV2 [62]. Therefore, BRD2 DNA hypomethylation and reduced expression in AD MG may contribute to Aβ plaque accumulation. This evidence further strengthened our hypothesis that DNA hypomethylation may be a critical mechanism compromising the ability of MG phagocytosis in AD pathology.

### Transcriptome signature of MG Aβ phagocytosis may contribute to human AD pathology

Our study used a comprehensive meta-analysis to analyze the expression of phagocytic genes in publicly available AD MG transcriptome datasets from AD patients and mouse AD models and experimental models of MG Aβ phagocytosis (Figs. 3 and 4). We identified 37 AD MG DEGs with a similar expression pattern in both species and 130 functional-validated Aβ phagocytic AD MG DEGs with consistent expression changes in AD MG (20 in human AD and 110 in mouse AD). Most of these genes and their expression pattern in AD MG provided the molecular signatures for a compensatory response of Aβ phagocytosis activation in human and mouse AD MG. Interestingly, we identified 14 Aβ phagocytic AD MG DEGs which exhibited reverse expression change in human AD MG versus experimental Aβ^+^ MG, which represent impaired MG Aβ phagocytosis signatures. However, such signature was absent in mouse AD MG (Supplemental Figure S1). This discrepancy between the mouse and human datasets might be due to disease staging in the human cases versus mice, as the AD phenotype develops around the ages of 80 years in human and between 6 and 15 months of age in mouse AD models. Based on this consideration, we proposed that the activation and impairment process of Aβ phagocytosis function may occur sequentially during AD development and that the impaired Aβ phagocytosis may impact the later stage of human AD development, contributing to the much more Aβ accumulation in the brain. To support this notion, we have identified 20 GWAS-mapped phagocytic AD MG DEGs (Supplemental Figure S2), which have been found to modulate the phagocytosis function in MG. Out of the 20 GWAS-mapped phagocytic AD MG DEGs, TREM2 (R47H substitution) was found to be associated with a predisposition to AD pathology by impairing Aβ phagocytosis [63–66]. In addition, a few other GWAS AD genes, such as CD33, MS4A, and APOE, were found to be associated with changes in MG phagocytic function in their mutated condition [67]. We believe that impaired Aβ clearance in MG is a significant contributor to AD development.

### Molecular targets responsible for HHcy-suppressed phagocytosis and HHcy-activated inflammatory response in AD MG

Finally, we identified 10 HHcy-altered phagocytic AD MG DEGs via overlapping analysis of the 353 HHcy MG DEGs with other MG DEGs related to AD phagocytosis and hypomethylation (Fig. 6). These findings led us to speculate two potential mechanisms underlying HHcy-exacerbated AD. Primarily, HHcy suppressed MG phagocytosis via upregulating Cacna2d4 and downregulating Flt1, Calponin 3, Igf1, and Celsr3. This was supported by previously reported functions implicated for these genes, such as Cacna2d4 in reducing Aβ clearance [68], Flt1 in mediating MG migration towards Aβ aggregates [69], Calponin 3 in regulating actin cytoskeleton rearrangement [70], Igf1 in promoting M2-like MΦ activation and phagocytic activity [71], and Celsr3 in binding oligomeric Aβ [70]. Another plausible compelling mechanism for HHcy-exacerbated AD is that HHcy promoted MG-derived inflammatory response via upregulating Ctse and downregulating Ch25h. It is reported that Ctse induced NF-κB activation for neurotoxic polarization of MG/MΦ [72] and that Ch25h catalyzed the formation of 25-hydroxycholesterol which suppresses interleukin-1-driven inflammation [73].

### Working models for HHcy-suppressed Aβ phagocytosis in AD MG

Aβ peptides are generated through the cleavage of APP by β-/γ-secretase, and then aggregate into Aβ fibril/plaque leading to AD development. MG primarily removes Aβ fibril/plaque via Aβ engulfment, phagosome formation, and lysosomal degradation. Our *Cbs*^*−*/*−*^ mice imitated human severe HHcy disease and presented hypomethylation status primarily due to elevated SAH levels, which resulted in protein/DNA hypomethylation. We identified 10 HHcy-altered phagocytic AD MG DEGs via screening *Cbs*^*−*/*−*^ MG transcriptome and combining with a series of comprehensive meta-analyses over a group of publicly available AD MG transcriptome databases. We proposed two working models for HHcy-suppressed Aβ phagocytosis in AD MG. Firstly, HHcy suppresses MG Aβ phagocytosis via impaired MG Aβ engulfment. Secondly, HHcy promoted inflammatory response via activating NF-κB and IL-1 pathways. We believe that hypomethylation is the key regulatory mechanism responsible for the transcriptional changes of these genes as elevated SAH levels and reduced SAM/SAH ratios have been confirmed in various experimental HHcy models by us and others [19, 27, 29, 30, 54]. In addition, elevated SAH levels were approved to trigger phagocytosis suppression in MΦ [74] and to activate NF-κB pathways and pro-inflammatory cytokine production in the endothelial cells [75]. This study only connected cacna2d4 induction with DNA hypomethylation in the human AD brain. This might be related to the small sample size limitation and experimental sensitivity in that study [51]. It is essential in the future research to evaluate DNA or protein methylation of targeted molecules and to characterize Hcy metabolism. Ongoing research in our laboratory is to validate mRNA changes and evaluate protein expression of the identified HHcy-altered phagocytic AD MG genes using ELISA and flow cytometry in the AD and HHcy mouse models.

## Conclusions

We have established sequential molecular signatures for a compensatory response of Aβ phagocytosis activation in human and mouse AD MG and an impaired Aβ phagocytosis in late-stage human AD MG. We demonstrated that hypomethylation is a potential regulatory mechanism mediating impaired MG Aβ phagocytosis in AD and HHcy. Investigating the mechanism underlying Aβ phagocytosis regulation in MG may lead to the discovery of novel therapeutic targets for AD.

### Supplementary Information

**Additional file 1: Supplemental Figure S1.** Functional-validated human/mouse Aβ phagocytic AD MG DEGs with opposing changes in gene expression in human/mouse AD MG and mouse Aβ+ MG (14 genes). A total of 14 functional-validated human Aβ phagocytic AD MG DEGs were identified by overlapping functional-validated human AD MG DEG with mouse Aβ+ MG screening dataset, displaying opposite changes in gene expression. Abbreviation: Aβ, amyloid beta; AD, Alzheimer’s disease; DEG, differentially expressed gene; FC, fold change; MG, microglia. **Supplemental Figure S2.** Identification of GWAS-mapped phagocytic AD MG DEGs altered by HHcy. (A) Identification of 431 GWAS-mapped AD genes. A summary diagram was created to establish a set of AD genes mapped by GWAS using a PubMed search. (B) GWAS-mapped AD MG DEGs (43). We identified 43 GWAS-mapped AD MG DEGs by overlapping 431 GWAS-mapped AD genes with 409 human and 777 mouse AD MG genes. Among the 43 genes, 4 were common to both species. We plotted the expression changes of these genes in AD MG using a histogram, where the dark red bars indicate genes that were identified in both human (C) and mouse (D) AD MG. (E) Network and neuro-immunological AD biological process. We used the 43 identified GWAS-mapped AD MG genes to perform Gene Ontology (GO) analysis and identify the top 15 neuro-immunological biological processes and molecular networks. (F) GWAS-mapped phagocytic AD MG DEGs altered by HHcy. GWAS-mapped phagocytic AD MG genes altered by HHcy were identified by overlapping 431 human GWAS-mapped AD genes with 326 phagocytic AD MG genes and 353 Cbs-/- MG genes. The functions and cellular locations of these genes are described in a table. Abbreviation: Aβ, amyloid beta; AD, Alzheimer’s disease; CI, confidence interval; DEG, differentially expressed gene; ER, endoplasmic reticulum; FC, fold change; FDR, false discovery rate; GO, gene ontology analysis; GWAS, Genome-wide association studies; HHcy, hyperhomocysteinemia; MG, microglia; PM, plasma membrane; TGN, trans-Golgi-network.

**Additional file 2: Supplemental Table 1:** Microglial differentially expressed genes (DEGs) of Cbs knockout versus control mice based on microglial bulk RNA-seq analysis. **Supplemental Table 2.** 409 AD MG DEGs from 4 human MG RNA-sequencing datasets (MG from AD patients, FC>=1.5, Adj p<0.05). **Supplemental Table 3.** 777 AD MG DEGs from 7 mouse MG RNA-sequencing datasets (MG from AD mice, FC>=1.5, Adj *p*< 0.05). **Supplemental Table 4.** 377 Mouse Aβ+ MG DEGs (Mouse Aβ+ MG screening, FC>=1.5, Adj *p*<0.05). **Supplemental Table 5.** 110 functional-validated mouse Aβ phagocytic AD MG DEGs. **Supplemental Table 6.** 286 MG genetic modifiers of phagocytosis (sgRNA library/Cas9 knockout phagocytic MG cell line screening).**Supplemental Table 7.** 2172 endocytosis-related genes involved in the biological processes of endocytosis, exocytosis, and transcytosis by searching public Mouse Genome Informatics and Gene Set Enrichment Analysis websites. **Supplemental Table 8.** 215 endocytosis-related AD MG DEGs by overlapping the 2172 endocytosis-related genes with identified human and mouse AD MG DEGs. **Supplemental Table 9.** total phagocytic AD MG DEGs (326 genes). **Supplemental Table 10.** A total of 431 human GWAS-mapped AD genes by reviewing literatures*. **Supplemental Table 11.** 559 differentially methylated/expressed genes (DM/EG) by examining AD DNA methylation and transcriptome dataset of 1030 healthy controls and AD patients.

## Data Availability

The data supporting the findings of this study are included in supplemental materials.

## Abbreviations

Aβ: Amyloid β
AD: Alzheimer’s disease
APP: Amyloid precursor protein
Cbs: Cystathionine-β-synthase
DEG: Differentially expressed gene
DM/EG: Differentially methylated and expressed genes
FC: Fold change
GO: Gene Ontology
GWAS: Genome-wide association studies
HHcy: Hyperhomocysteinemia
HM: Homocysteine-methionine
IPA: Ingenuity pathway analysis
KEGG: Kyoto Encyclopedia of Genes and Genomes
MC: Monocyte
MG: Microglia
MΦ: Macrophages
SAH: S-Adenosylhomocysteine
SAM: S-Adenosylmethionine
Sc: Single cell
Sn: Single nucleus
PCP: Planar cell polarity
