# Supplementary material for: Identification of methylation-regulated genes modulating microglial phagocytosis in hyperhomocysteinemia-exacerbated Alzheimer’s disease
Source: Alzheimers Res Ther. 2023 Oct 3;15:164. doi: 10.1186/s13195-023-01311-9 (PMC10546779; doi:10.1186/s13195-023-01311-9)
Supplement: Supplementary file 2 — Additional file 2: Supplemental Table 1: Microglial differentially expressed genes (DEGs) of Cbs knockout versus control mice based on microglial bulk RNA-seq analysis. Supplemental Table 2. 409 AD MG DEGs from 4 human MG RNA-sequencing datasets (MG from AD patients, FC>=1.5, Adj p<0.05). Supplemental Table 3. 777 AD MG DEGs from 7 mouse MG RNA-sequencing datasets (MG from AD mice, FC>=1.5, Adj p< 0.05). Supplemental Table 4. 377 Mouse Aβ+ MG DEGs (Mouse Aβ+ MG screening, FC>=1.5, Adj p<0.05). Supplemental Table 5. 110 functional-validated mouse Aβ phagocytic AD MG DEGs. Supplemental Table 6. 286 MG genetic modifiers of phagocytosis (sgRNA library/Cas9 knockout phagocytic MG cell line screening).Supplemental Table 7. 2172 endocytosis-related genes involved in the biological processes of endocytosis, exocytosis, and transcytosis by searching public Mouse Genome Informatics and Gene Set Enrichment Analysis websites. Supplemental Table 8. 215 endocytosis-related AD MG DEGs by overlapping the 2172 endocytosis-related genes with identified human and mouse AD MG DEGs. Supplemental Table 9. total phagocytic AD MG DEGs (326 genes). Supplemental Table 10. A total of 431 human GWAS-mapped AD genes by reviewing literatures*. Supplemental Table 11. 559 differentially methylated/expressed genes (DM/EG) by examining AD DNA methylation and transcriptome dataset of 1030 healthy controls and AD patients. [file 13195_2023_1311_MOESM2_ESM.pptx]

## Slide 1
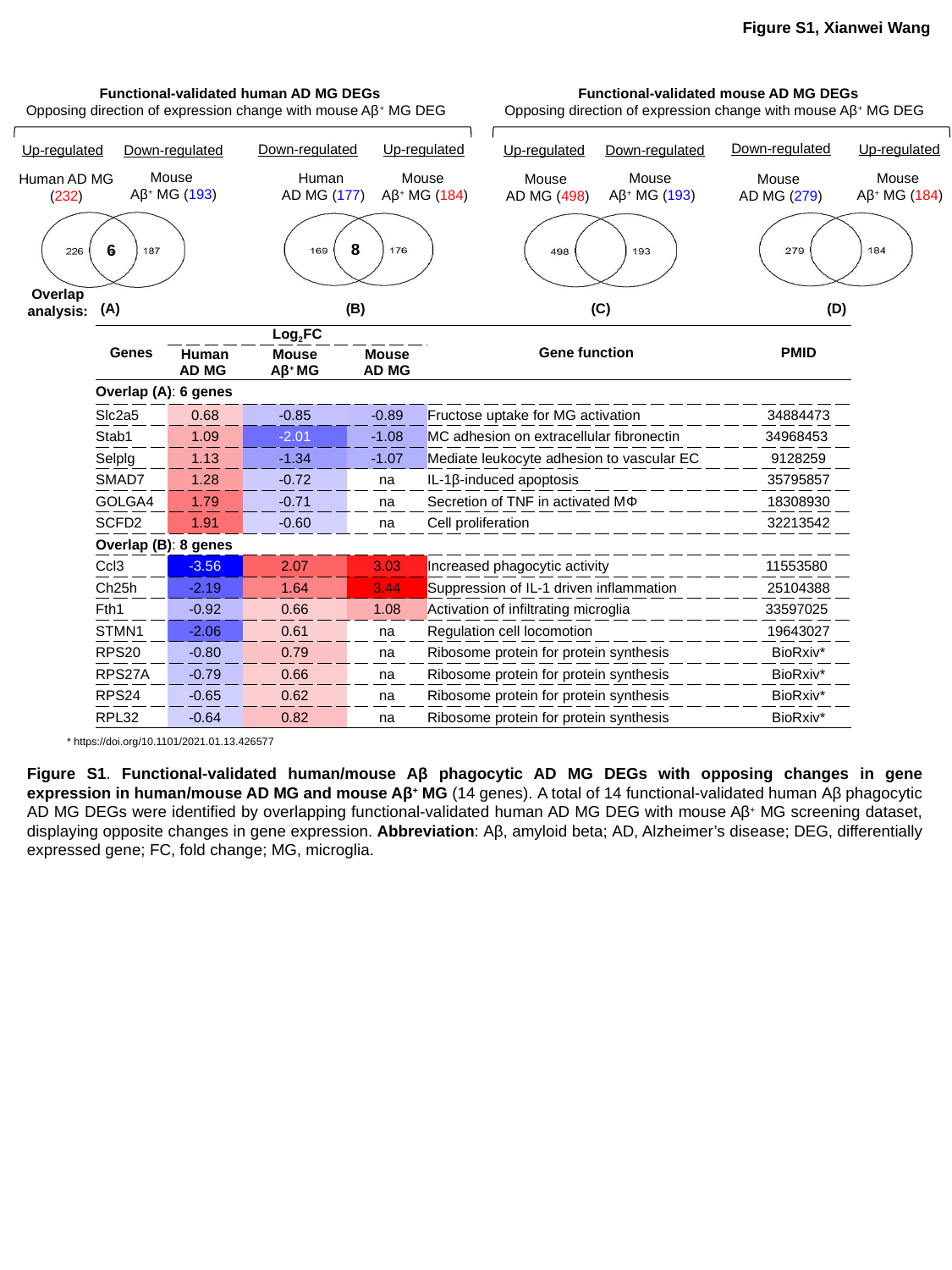

Figure S1, Xianwei Wang
Functional-validated human AD MG DEGs
Opposing direction of expression change with mouse Aβ+ MG DEG
Functional-validated mouse AD MG DEGs
Opposing direction of expression change with mouse Aβ+ MG DEG
Down-regulated
Up-regulated
Up-regulated
Up-regulated
Down-regulated
Down-regulated
Down-regulated
Up-regulated
Mouse
Aβ+ MG (193)
Mouse
Aβ+ MG (184)
Human AD MG
(232)
Human
AD MG (177)
Mouse
Aβ+ MG (184)
Mouse
Aβ+ MG (193)
Mouse
AD MG (279)
Mouse
AD MG (498)
8
6
Overlap analysis:
(A)
(B)
(C)
(D)
| Genes | Log2FC | | | Gene function | PMID |
| --- | --- | --- | --- | --- | --- |
| | Human AD MG | Mouse Aβ+ MG | Mouse AD MG | | |
| Overlap (A): 6 genes | | | | | |
| Slc2a5 | 0.68 | -0.85 | -0.89 | Fructose uptake for MG activation | 34884473 |
| Stab1 | 1.09 | -2.01 | -1.08 | MC adhesion on extracellular fibronectin | 34968453 |
| Selplg | 1.13 | -1.34 | -1.07 | Mediate leukocyte adhesion to vascular EC | 9128259 |
| SMAD7 | 1.28 | -0.72 | na | IL-1β-induced apoptosis | 35795857 |
| GOLGA4 | 1.79 | -0.71 | na | Secretion of TNF in activated MΦ | 18308930 |
| SCFD2 | 1.91 | -0.60 | na | Cell proliferation | 32213542 |
| Overlap (B): 8 genes | | | | | |
| Ccl3 | -3.56 | 2.07 | 3.03 | Increased phagocytic activity | 11553580 |
| Ch25h | -2.19 | 1.64 | 3.44 | Suppression of IL-1 driven inflammation | 25104388 |
| Fth1 | -0.92 | 0.66 | 1.08 | Activation of infiltrating microglia | 33597025 |
| STMN1 | -2.06 | 0.61 | na | Regulation cell locomotion | 19643027 |
| RPS20 | -0.80 | 0.79 | na | Ribosome protein for protein synthesis | BioRxiv\* |
| RPS27A | -0.79 | 0.66 | na | Ribosome protein for protein synthesis | BioRxiv\* |
| RPS24 | -0.65 | 0.62 | na | Ribosome protein for protein synthesis | BioRxiv\* |
| RPL32 | -0.64 | 0.82 | na | Ribosome protein for protein synthesis | BioRxiv\* |
* https://doi.org/10.1101/2021.01.13.426577
Figure S1. Functional-validated human/mouse Aβ phagocytic AD MG DEGs with opposing changes in gene expression in human/mouse AD MG and mouse Aβ+ MG (14 genes). A total of 14 functional-validated human Aβ phagocytic AD MG DEGs were identified by overlapping functional-validated human AD MG DEG with mouse Aβ+ MG screening dataset, displaying opposite changes in gene expression. Abbreviation: Aβ, amyloid beta; AD, Alzheimer’s disease; DEG, differentially expressed gene; FC, fold change; MG, microglia.

## Slide 2
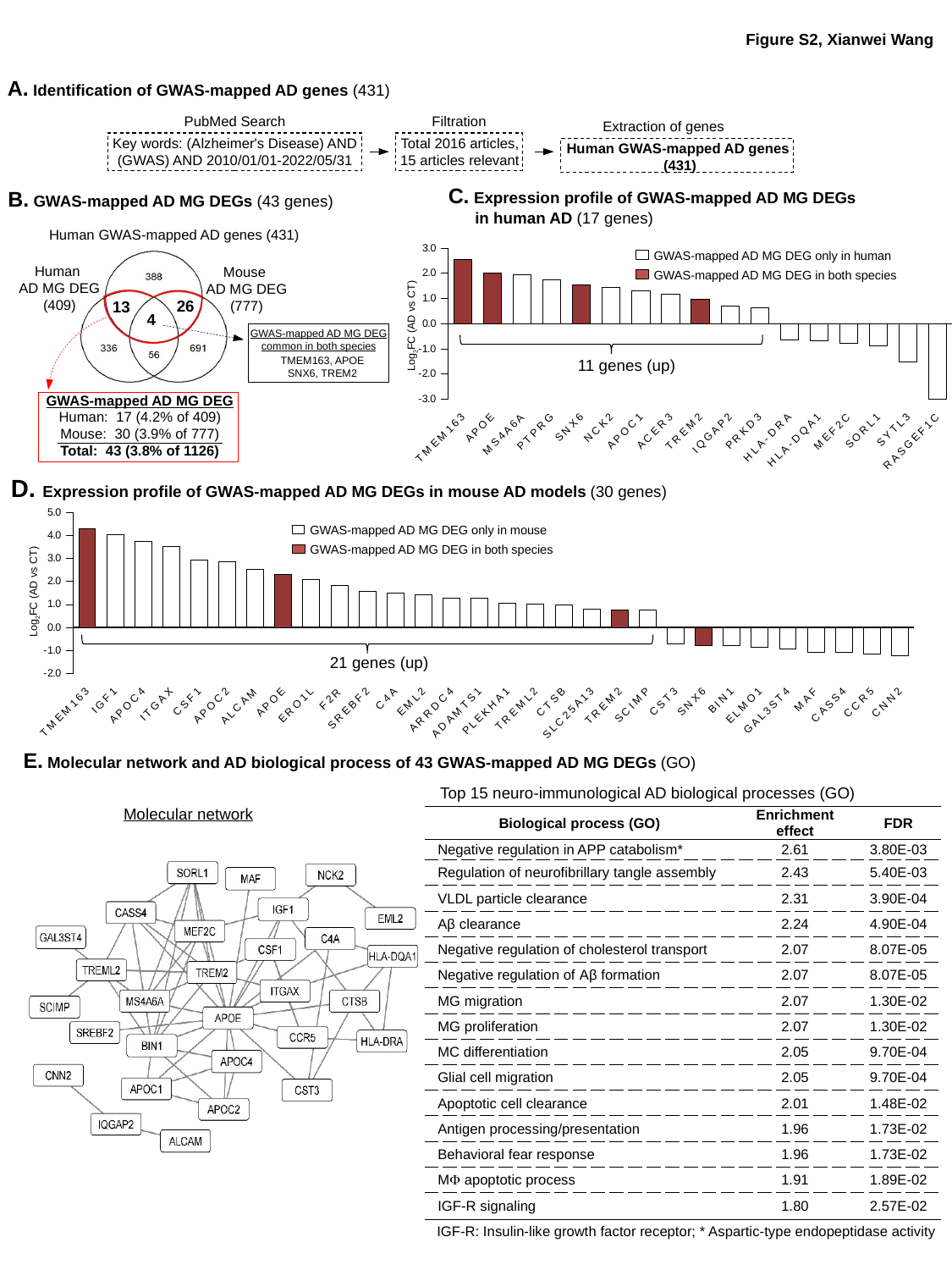

Figure S2, Xianwei Wang
A. Identification of GWAS-mapped AD genes (431)
PubMed Search
Filtration
Extraction of genes
Key words: (Alzheimer's Disease) AND (GWAS) AND 2010/01/01-2022/05/31
Total 2016 articles,
15 articles relevant
Human GWAS-mapped AD genes
 (431)
B. GWAS-mapped AD MG DEGs (43 genes)
C. Expression profile of GWAS-mapped AD MG DEGs
 in human AD (17 genes)
### Chart
| Category | hAvLogFC |
|---|---|
| TMEM163 | 2.5538616 |
| APOE | 2.0096148 |
| MS4A6A | 1.946685 |
| PTPRG | 1.74661319874638 |
| SNX6 | 1.526 |
| NCK2 | 1.4436921 |
| APOC1 | 1.30413919241884 |
| ACER3 | 1.1860811 |
| TREM2 | 0.951750029823305 |
| IQGAP2 | 0.6927 |
| PRKD3 | 0.6424 |
| HLA-DRA | -0.657 |
| HLA-DQA1 | -0.6852 |
| MEF2C | -0.7651 |
| SORL1 | -0.8884231 |
| SYTL3 | -1.51435684701517 |
| RASGEF1C | -3.0014677 |GWAS-mapped AD MG DEG only in human
GWAS-mapped AD MG DEG in both species
Log2FC (AD vs CT)
Human GWAS-mapped AD genes (431)
26
13
4
Human
AD MG DEG
(409)
Mouse
AD MG DEG
(777)
GWAS-mapped AD MG DEG
common in both species
TMEM163, APOE
SNX6, TREM2
GWAS-mapped AD MG DEG
Human: 17 (4.2% of 409)
Mouse: 30 (3.9% of 777)
Total: 43 (3.8% of 1126)
11 genes (up)
D. Expression profile of GWAS-mapped AD MG DEGs in mouse AD models (30 genes)
### Chart
| Category | mAvLogFC |
|---|---|
| TMEM163 | 4.30229963158545 |
| IGF1 | 4.04256774555375 |
| APOC4 | 3.7312439975 |
| ITGAX | 3.53191453776787 |
| CSF1 | 2.93737051298674 |
| APOC2 | 2.87545268366667 |
| ALCAM | 2.523704309 |
| APOE | 2.32847886924748 |
| ERO1L | 2.07828858966667 |
| F2R | 1.818434478 |
| SREBF2 | 1.55590370153496 |
| C4A | 1.5025 |
| EML2 | 1.4391522845 |
| ARRDC4 | 1.29258910597092 |
| ADAMTS1 | 1.2725 |
| PLEKHA1 | 1.05001968789265 |
| TREML2 | 1.031046251 |
| CTSB | 1.00000948447952 |
| SLC25A13 | 0.8186262445 |
| TREM2 | 0.781376922348327 |
| SCIMP | 0.753635215 |
| CST3 | -0.6972208265 |
| SNX6 | -0.789342439638774 |
| BIN1 | -0.8021031975 |
| ELMO1 | -0.875499477690413 |
| GAL3ST4 | -0.9375351415 |
| MAF | -1.06652522246046 |
| CASS4 | -1.08128522 |
| CCR5 | -1.14173140493169 |
| CNN2 | -1.23341081325 |GWAS-mapped AD MG DEG only in mouse
GWAS-mapped AD MG DEG in both species
Log2FC (AD vs CT)
21 genes (up)
E. Molecular network and AD biological process of 43 GWAS-mapped AD MG DEGs (GO)
Top 15 neuro-immunological AD biological processes (GO)
Molecular network
| Biological process (GO) | Enrichment effect | FDR |
| --- | --- | --- |
| Negative regulation in APP catabolism\* | 2.61 | 3.80E-03 |
| Regulation of neurofibrillary tangle assembly | 2.43 | 5.40E-03 |
| VLDL particle clearance | 2.31 | 3.90E-04 |
| Aβ clearance | 2.24 | 4.90E-04 |
| Negative regulation of cholesterol transport | 2.07 | 8.07E-05 |
| Negative regulation of Aβ formation | 2.07 | 8.07E-05 |
| MG migration | 2.07 | 1.30E-02 |
| MG proliferation | 2.07 | 1.30E-02 |
| MC differentiation | 2.05 | 9.70E-04 |
| Glial cell migration | 2.05 | 9.70E-04 |
| Apoptotic cell clearance | 2.01 | 1.48E-02 |
| Antigen processing/presentation | 1.96 | 1.73E-02 |
| Behavioral fear response | 1.96 | 1.73E-02 |
| M apoptotic process | 1.91 | 1.89E-02 |
| IGF-R signaling | 1.80 | 2.57E-02 |
IGF-R: Insulin-like growth factor receptor; * Aspartic-type endopeptidase activity

## Slide 3
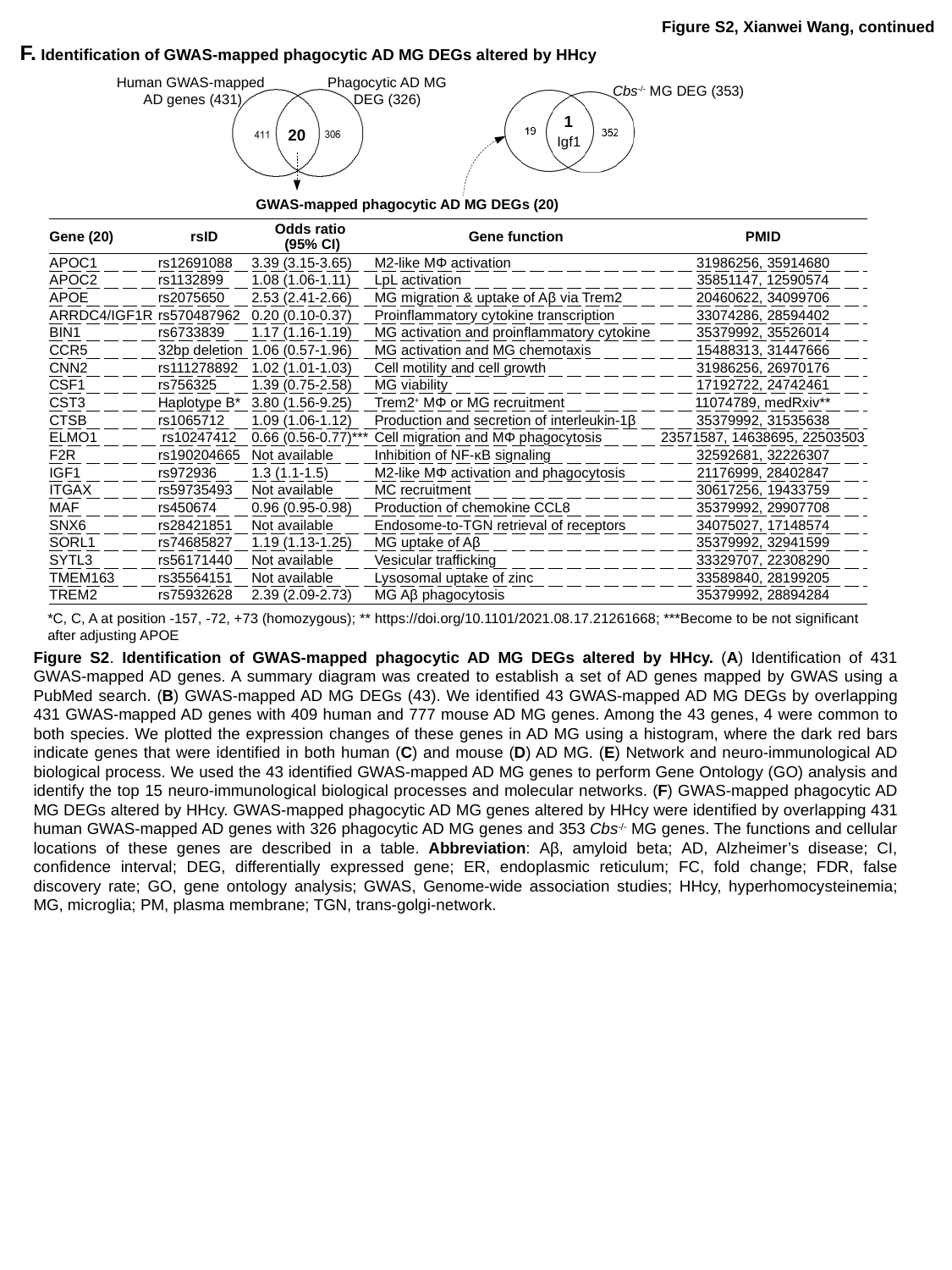

Figure S2, Xianwei Wang, continued
F. Identification of GWAS-mapped phagocytic AD MG DEGs altered by HHcy
Human GWAS-mapped AD genes (431)
Phagocytic AD MG DEG (326)
Cbs-/- MG DEG (353)
20
1
Igf1
GWAS-mapped phagocytic AD MG DEGs (20)
| Gene (20) | rsID | Odds ratio (95% CI) | Gene function | PMID |
| --- | --- | --- | --- | --- |
| APOC1 | rs12691088 | 3.39 (3.15-3.65) | M2-like MΦ activation | 31986256, 35914680 |
| APOC2 | rs1132899 | 1.08 (1.06-1.11) | LpL activation | 35851147, 12590574 |
| APOE | rs2075650 | 2.53 (2.41-2.66) | MG migration & uptake of Aβ via Trem2 | 20460622, 34099706 |
| ARRDC4/IGF1R | rs570487962 | 0.20 (0.10-0.37) | Proinflammatory cytokine transcription | 33074286, 28594402 |
| BIN1 | rs6733839 | 1.17 (1.16-1.19) | MG activation and proinflammatory cytokine | 35379992, 35526014 |
| CCR5 | 32bp deletion | 1.06 (0.57-1.96) | MG activation and MG chemotaxis | 15488313, 31447666 |
| CNN2 | rs111278892 | 1.02 (1.01-1.03) | Cell motility and cell growth | 31986256, 26970176 |
| CSF1 | rs756325 | 1.39 (0.75-2.58) | MG viability | 17192722, 24742461 |
| CST3 | Haplotype B\* | 3.80 (1.56-9.25) | Trem2+ MΦ or MG recruitment | 11074789, medRxiv\*\* |
| CTSB | rs1065712 | 1.09 (1.06-1.12) | Production and secretion of interleukin-1β | 35379992, 31535638 |
| ELMO1 | rs10247412 | 0.66 (0.56-0.77)\*\*\* | Cell migration and MΦ phagocytosis | 23571587, 14638695, 22503503 |
| F2R | rs190204665 | Not available | Inhibition of NF-κB signaling | 32592681, 32226307 |
| IGF1 | rs972936 | 1.3 (1.1-1.5) | M2-like MΦ activation and phagocytosis | 21176999, 28402847 |
| ITGAX | rs59735493 | Not available | MC recruitment | 30617256, 19433759 |
| MAF | rs450674 | 0.96 (0.95-0.98) | Production of chemokine CCL8 | 35379992, 29907708 |
| SNX6 | rs28421851 | Not available | Endosome-to-TGN retrieval of receptors | 34075027, 17148574 |
| SORL1 | rs74685827 | 1.19 (1.13-1.25) | MG uptake of Aβ | 35379992, 32941599 |
| SYTL3 | rs56171440 | Not available | Vesicular trafficking | 33329707, 22308290 |
| TMEM163 | rs35564151 | Not available | Lysosomal uptake of zinc | 33589840, 28199205 |
| TREM2 | rs75932628 | 2.39 (2.09-2.73) | MG Aβ phagocytosis | 35379992, 28894284 |
*C, C, A at position -157, -72, +73 (homozygous); ** https://doi.org/10.1101/2021.08.17.21261668; ***Become to be not significant after adjusting APOE
Figure S2. Identification of GWAS-mapped phagocytic AD MG DEGs altered by HHcy. (A) Identification of 431 GWAS-mapped AD genes. A summary diagram was created to establish a set of AD genes mapped by GWAS using a PubMed search. (B) GWAS-mapped AD MG DEGs (43). We identified 43 GWAS-mapped AD MG DEGs by overlapping 431 GWAS-mapped AD genes with 409 human and 777 mouse AD MG genes. Among the 43 genes, 4 were common to both species. We plotted the expression changes of these genes in AD MG using a histogram, where the dark red bars indicate genes that were identified in both human (C) and mouse (D) AD MG. (E) Network and neuro-immunological AD biological process. We used the 43 identified GWAS-mapped AD MG genes to perform Gene Ontology (GO) analysis and identify the top 15 neuro-immunological biological processes and molecular networks. (F) GWAS-mapped phagocytic AD MG DEGs altered by HHcy. GWAS-mapped phagocytic AD MG genes altered by HHcy were identified by overlapping 431 human GWAS-mapped AD genes with 326 phagocytic AD MG genes and 353 Cbs-/- MG genes. The functions and cellular locations of these genes are described in a table. Abbreviation: Aβ, amyloid beta; AD, Alzheimer’s disease; CI, confidence interval; DEG, differentially expressed gene; ER, endoplasmic reticulum; FC, fold change; FDR, false discovery rate; GO, gene ontology analysis; GWAS, Genome-wide association studies; HHcy, hyperhomocysteinemia; MG, microglia; PM, plasma membrane; TGN, trans-golgi-network.
